# Delirium with Concurrent Use of Lithium and ECT and the Safety Implications: Case Reports and Review of the Literature

**DOI:** 10.1155/2023/9117292

**Published:** 2023-05-09

**Authors:** Mustafa Ali, Barikar C. Malathesh, Seshadri Sekhar Chatterjee, Soumitra Das, Prakriti Pokhrel, Mary Elizabeth Trejo Hernandez, John C. Murnin

**Affiliations:** ^1^Government Medical College, Jammu and Kashmir, India; ^2^All India Institute of Medical Sciences, Bibinagar, India; ^3^Queensland Health, Australia; ^4^Royal Melbourne Hospital, Australia; ^5^Kathmandu Medical College Teaching Hospital, Sinamangal, Nepal; ^6^Universidad Autonoma de Guadalajara School of Medicine, Mexico; ^7^Burrell College of Osteopathic Medicine, New Mexico, USA

## Abstract

Using electroconvulsive treatment and lithium together to treat acute manic episodes is common, but the effects of combining these therapies vary according to the literature. Some studies have found severe adverse side effects, while others have found the combination of both medications safe and helpful. To investigate potential adverse side effects, this study reports on two cases where bipolar affective disorder patients developed delirium after receiving electroconvulsive therapy and lithium concurrently. The delirium was attributed only to the combined administration of these medicines after ruling out other potential causes. Additionally, alterations in blood-brain barrier permeability, such as those caused by electroconvulsive therapy and age, increased the likelihood of delirium. As a result, caution should be taken when using this combination of medicines, especially in those predisposed to delirium. This study established links between these medications and adverse effects, such as delirium. Further research is necessary to determine the efficacy and risks of combining these medications, establish causality, and develop prevention strategies.

## 1. Introduction

Lithium is the first-choice treatment for bipolar disorder with mania [1] and is also effective for unipolar depression and reducing suicidal behavior [2]. If medication alone is insufficient for these patients, electroconvulsive therapy (ECT) may be recommended [2]. ECT is proven effective for bipolar depression, mania, and mixed affective states [3–5] and may also be used as a maintenance therapy [6, 7]. Since ECT is usually considered after other medications have been tried, including lithium, many who receive ECT have previously tried other treatments.

Using ECT while patients are taking lithium is a common practice in the healthcare industry [8], but the literature on this combination offers mixed results and no clear consensus [9, 10]. Some studies have shown that combining lithium and ECT may result in issues such as prolonged seizures [6, 11–14], prolonged apnea [9], serotonin syndrome with focal seizures [13], delirium [5, 6, 15], and declining cognitive abilities [14]. However, a few studies have found no adverse effects when using ECT and lithium together [9]. Phase 2 of the Prolonging Remission in Depressed Elderly (PRIDE) study reported no significant side effects in geriatric patients who received a combination of ECT and venlafaxine and lithium regimen in phase 2 [16]. A prospective study on this combination found no differences in seizure characteristics, apnea time, or anesthetic recovery [17].

Despite the studies suggesting varied outcomes with the concurrent use of lithium and ECT, several authors have proposed avoiding the combination. However, abrupt discontinuation of lithium has negative implications, such as potential resistance to lithium, worsening symptoms, or increased suicide ideation [18–20]. Considering these cases, psychiatrists may continue using ECT on patients currently taking lithium.

In the background of the above literature, we present two cases who developed delirium after receiving a combination of ECT and lithium. We also review similar cases from the literature, including managing such cases.

## 2. Case Reports

### 2.1. Case 1

A 28-year-old man with bipolar affective disorder presented to a psychiatrist with symptoms of mania and psychosis, characterized by pervasive elated mood and irritability, increased psychomotor activity, pressured speech, delusions of grandiosity, decreased need for sleep, and disinhibition. The patient had experienced two episodes of mania in the past eight years, both due to noncompliance with medication. The current episode lasted 15 days and was caused by not taking medication. The patient responded well to the lithium 900 mg/d and risperidone 4 mg/day combination along with ECT in the previous inpatient psychiatric hospitalization. However, after a week of treatment with lithium 900 mg/d (serum lithium 0.87 mEq/L) and risperidone 4 mg/d, he continued to be symptomatic with a YMRS score of 36. Therefore, ECT was initiated as an augmenting agent, starting with a modified, bitemporal, two times suprathreshold, and thrice-weekly schedule. After the fourth ECT, the patient's symptoms began to improve. However, after the fifth ECT, he developed delirium, characterized by disorientation to time and place, visuospatial disorientation, impaired attention and concentration, immediate impaired recall, and recent memory and sleep disturbance. Complete neurological and blood investigations were done to rule out any other cause for delirium. Blood investigations were normal. No other apparent cause was found for the delirium attributed to ECT, which was hence stopped. The patient's delirium resolved over ten days, and he was managed with lithium 1050 mg/d, risperidone 6 mg/d, trihexyphenidyl 2 mg, and clonazepam 1 mg/d. The patient improved in affective and psychotic symptoms over the next two weeks, and the YMRS score came down to 12.

### 2.2. Case 2

A man aged 25 had bipolar affective disorder with a current episode of mania, which included an elated mood, talking excessively, logorrhea, and a reduced need for sleep. He was taking a combination of lithium (900 mg/d) and risperidone (8 mg/day) but still had significant problems in his personal, social, and work lives. He decided to lower the lithium dose himself from 900 to 600 mg, leading to his current episode. His serum lithium level was 0.32 mEq/L when he was admitted to the hospital. He remained symptomatic despite increasing the dose to 900 mg/day. Since he had responded well to ECT without any adverse effects in the past with no complications, it was considered a treatment option to augment his current therapy. He received modified, bifrontal, suprathreshold ECT on alternate days, which led to improvement in his symptoms. However, after the 6th ECT, he developed delirium symptoms, including confusion about time, place, and person. Blood tests showed normal results with a serum lithium level of 0.82 mEq/L. These symptoms lasted for 15 days after stopping ECT. After this, he was treated with lithium 900 mg/d, risperidone 8 mg/d, and lorazepam 4 mg/d for one week before discharge.

## 3. Discussions

The literature on lithium-ECT coadministration mainly consists of case reports and retrospective studies. A prospective randomized study by Coppen and Abou-Saleh emphasized prophylaxis against recurrent mood episodes but did not incorporate assessments of neurologic examinations, seizure duration, postictal orientation, or spontaneous breathing time [21]. Thirthalli et al. compared seizure parameters, succinylcholine interaction, cardiovascular response, ECT recovery, and complications in patients with or without lithium therapy. The groups showed no significant difference in seizure variables, apnea time, or anesthesia recovery. However, serum lithium levels and concurrent use of lithium were positively associated with the duration of post-ECT recovery. Lithium use during ECT was considered safe within therapeutic range limits. Limitations include nonrandomization and a younger patient population (mean age 26 for the lithium group vs. 29.78 for the nonlithium group) without comorbidities [17]. Other studies which reported the safety of concurrent use of lithium and ECT without any neurological adverse effects are Dolenc and Rasmussen and phase 2 of the PRIDE study. Dolenc and Rasmussen reported 12 cases where ECT and lithium were safely combined without neurological adverse effects [9]. Phase 2 of the PRIDE study found no significant side effects in geriatric individuals who received a combination of ECT and a venlafaxine and lithium regimen [16].

Despite prior studies showing no neurological adverse effects from combining lithium and ECT, other case reports and retrospective studies suggest a higher incidence of delirium.  Table 1 summarizes evidence of a negative interaction between the two, emphasizing delirium. Various hypotheses have been proposed to explain this phenomenon; for instance, ECT may increase the permeability of the blood-brain barrier (BBB), thereby increasing cerebral blood flow [22]. This dysfunction may cause a sudden increase in lithium ions entering the brain or a redistribution of concentrations within cerebral tissue compartments, resulting in peak levels observed in white matter [23]. Age-related factors can influence BBB permeability fluctuations as well as chemical means [24] in human subjects. ECT and lithium have been known to trigger seizures, exert anticonvulsant effects, and induce convulsive effects [14]. These substances' combined impacts may exceed tolerance thresholds or disrupt opposing mechanisms, leading to serum levels exceeding 0.8 mEq/L, as demonstrated above.

Individuals with diffuse encephalopathy or degenerative brain disorder may be more susceptible to the adverse effects of electroconvulsive therapy. Patients with preexisting organic brain injury are at higher risk for cognitive side effects from lithium and ECT. After analysis, Rudorfer et al. found no discernible factors contributing to the observed toxicity levels in terms of pharmacokinetics or drug interactions. [25]. However, El-Mallakh presented a different viewpoint and came to the conclusion that ECT could augment the central manifestation of lithium poisoning [26]. Husain et al. further suggested that ECT and lithium may have an additive effect on the noradrenergic system that would alter epileptogenicity [6]. Awasthi et al. observed increased noradrenaline uptake in mouse brains after the electroconvulsive shock (ECS) series, although they attributed these findings to BBB alterations [27]. In animal models, El-MAllakh found that concurrent lithium and ECS treatment failed to demonstrate a significant elevation of brain lithium concentration (nor brain to serum concentration) compared to lithium treatment alone. However, the study did not investigate intracellular lithium levels [26].

ECT may cause delirium, but only in some cases. Therefore, identifying predictors of delirium is essential. Research suggests that patients with depressive episodes are more likely to develop delirium than those with manic episodes when taking mood stabilizers and antipsychotics. Patel et al.'s subanalysis found that the prevalence of delirium was 7.8% in MDD patients, 3.4% in bipolar depression patients, and 0% in bipolar mania patients [8]. This could be due to differences in metabolism or tolerance levels for lithium between the two disorders or a function of lithium dosage; higher doses increase the risk of developing delirium.

According to Reti et al. [45], the duration of seizures is indicative of delirium following electroconvulsive therapy, while Jo et al. [43] found that catatonic symptoms before ECT serve as predictors for post-ECT delirium. Additionally, certain anesthetics administered during ECT like etomidate have been linked to an increased likelihood of developing delirium [46]. It would be beneficial for future research endeavors to explore the impact of electrode placement on the occurrence of delirium in individuals undergoing ECT.

Thus, the studies discussed above imply that ECT as well as age are risk factors for a change in BBB permeability to certain medications or molecules.

## 4. Conclusions

Previous studies on the risks involved in combining lithium and ECT have mostly been case reports and series which lack proper control. Using this combination on elderly patients or those with preexisting brain lesions requires extreme caution. Reducing the dose of lithium or discontinuing its use during ECT can minimize adverse effects, and a risk-benefit analysis is recommended. Recognizing the possible unfavorable side effects when using both therapies is vital. The two case studies demonstrate that combining ECT and lithium to treat acute manic episodes can cause delirium. The cause of delirium in both cases was thoroughly examined and attributed to the concurrent use of these therapies. Further studies are needed to ascertain the effectiveness and risks of combining ECT and lithium.

## Figures and Tables

**Table 1:** Lithium-ECT induced delirium.

Sl no	Author(s) and year	Type of study (no. of cases)	Serum lithium	Comments
1	Jephcott and Kerry (1974) [28]	Case report (1)	3.4 mEq/L	Developed somnolence and lethargy after 2nd ECT [28]
2	Ray (1975) [29]	Case report (1)	0.46 mEq/L	Developed delirium after 3rd ECT [29]
3	Hoenig and Chaulk (1977) [30]	Case report (1)	1.09 mEq/L	Developed severe confusion after 1st ECT [30]
4	Zwil et al. [31]	Case report (1)	NA	Developed confusion and cognitive impairment [31]
5	Remick (1978) [32]	Case report (1)	1.0	Developed delirium after 4th ECT [32]
6	Mandel et al. (1980) [33]	Case report (2)	0.98 mEq/L and 0.60 mEq/L	(a) Developed prolonged confusion after 3rd ECT (b) Developed confusion after 2nd ECT [33]
7	Weiner et al. (1980) [34]	Case report (1)	0.37 mEq/L	Developed delirium after 5th ECT [34]
8	Small et al. [14]	Retrospective Chart Review (25)	NA	ECT/Li patients compared with age-matched controls receiving ECT without Li. The combination group had more confusion and memory impairment on chart review [14]
9	De Paulo et al. [35]	Case report (1)	0.8 mEq/L	Li started after 4th ECT—delirium ensued [35]
10	Ahmed and Stein (1987) [36]	Case report (1)	Normal S.Li level	Developed confusion after 2nd ECT [36]
11	Sartorius et al. [13]	Chart review (7)	NA	1 patient developed prolonged disorientation after ECT [13]
12	Penney et al. (1990) [37]	Chart review (27)	NA	Patients in the ECT+Li groups developed more post ECT confusion than the ECT group [37]
13	Jha AK (1996) [38]	Retrospective case-control (31)	NA	10% of patients developed delirium [38]
14	Stewart [39]	Case report (1)	NA	Developed increase in confusion while on combination of ECT and lithium [39]
15	Hassamal et al. (2013) [40]	Case report (1)	<0.3 mEq/L	Developed delirium after 3rd ECT [40]
16	Sadananda et al. (2013) [15]	Case report (1)	0.9 mEq/L	Developed delirium after 6th ECT [15]
17	Hategan and Bourgeois (2014) [41]	Case report (1)	0.3 mEq/L	Developed delirium after 1st and 2nd ECT [41]
18	Deuschle et al. [42]	Case report (1)	0.12 mEq/L	Developed delirium 2 days after starting lithium after 5th ECT [42]
19	Patel et al. [8]	Retrospective case-control (34)	NA	5.7% of patients developed acute delirium 2.4% of patients developed cognitive impairment [8]
20	Jo et al. (2021) [43]	Retrospective chart review (1)	NA	1 of 6 patients on lithium developed acute delirium after ECT [43]
21	Yang et al. (2022) [44]	Case report (1)	1.21 mEq/L	Developed delirium after 9th ECT [44]
